# Role of tube current variation and metal artifact reduction tool in CBCT for detection of missing MB2 canal

**DOI:** 10.1590/0103-644020256491

**Published:** 2025-10-24

**Authors:** Caio Alencar-Palha, Marcyele Natane da Silva Morais, Fernanda Bulhões Fagundes, Lucas P. Lopes Rosado, Deborah Queiroz Freitas, Frederico Sampaio Neves

**Affiliations:** 1 Department of Oral Diagnosis, Piracicaba Dental School, University of Campinas, Piracicaba, SP, Brazil; 2 Department of Dentistry, University Center of Espírito Santo (UNESC), Colatina, ES, Brazil; 3 Department of Propaedeutics and Integrated Clinic, Division of Oral Radiology, School of Dentistry, Federal University of Bahia, Salvador, BA, Brazil

**Keywords:** Mesiobuccal canal, cone beam computed tomography, noise, artifacts, radiation dose

## Abstract

We aimed to evaluate the effect of varying the tube current and the Metal Artifact Reduction (MAR) tool on the detection of the missing second mesiobuccal (MB2) canal. Forty maxillary molars (20 with and 20 without MB2) were selected and confirmed by micro-CT. The teeth underwent endodontic preparation, with the canals filled using gutta-percha and a silver-palladium metal post. CBCT scans were acquired using the OP300 Maxio system (voxel size: 0.085 mm, FOV: 50 x 50 mm) at 4 mA, 6.3 mA, and 12.5 mA, with and without MAR activation. The areas under the receiver operating characteristic curves (AUCs), sensitivities, and specificities were calculated for five examiners and compared using two-way ANOVA (p < 0.05). Intra- and interexaminer agreements were assessed using the weighted Kappa test. The AUC values ranged from 0.90 to 0.94, sensitivity from 0.75 to 0.78, and specificity from 0.89 to 0.95. Intra- and interexaminer indices ranged from moderate to substancial. Based on the tube current settings or MAR activation, there were no significant differences in the MB2 canal detection. Therefore, varying the tube current and MAR tool activation did not affect MB2 canal detection, with values close to 4 mA without MAR activation recommended in this scenario to reflect a better dose-benefit ratio.

## Resumo

Nosso objetivo foi avaliar o efeito da variação da corrente do tubo e da ferramenta *Metal Artifact Reduction* (MAR) na detecção do segundo canal mesiovestibular (MV2) não tratado. Quarenta molares superiores (20 com e 20 sem MV2) foram selecionados e confirmados por micro-CT. Os dentes foram submetidos ao preparo endodôntico, com canais preenchidos com guta-percha e um pino metálico de prata-paládio. Os exames de TCFC foram adquiridos usando o sistema OP300 Maxio (tamanho do voxel: 0,085 mm, FOV: 50x50 mm) a 4 mA, 6,3 mA e 12,5 mA, com e sem ativação da MAR. A área sob a curva ROC, a sensibilidade e a especificidade foram calculadas para cinco examinadores e comparadas usando ANOVA de dois fatores (p<0,05). As concordâncias intra e interexaminadores foram avaliadas usando o teste Kappa ponderado. Os valores de AUC variaram de 0,90 a 0,94, a sensibilidade de 0,75 a 0,78 e a especificidade de 0,89 a 0,95. Os índices intra e interexaminadores variaram de moderados a substanciais. Com base nas configurações de corrente do tubo ou na ativação da MAR, não houve diferenças significativas na detecção do canal MV2. Portanto, a variação da corrente do tubo e a ativação da ferramenta MAR não afetaram a sua detecção, com valores próximos a 4 mA sem ativação do MAR recomendados nesse cenário para refletir uma melhor relação dose-benefício.

**Figure:**
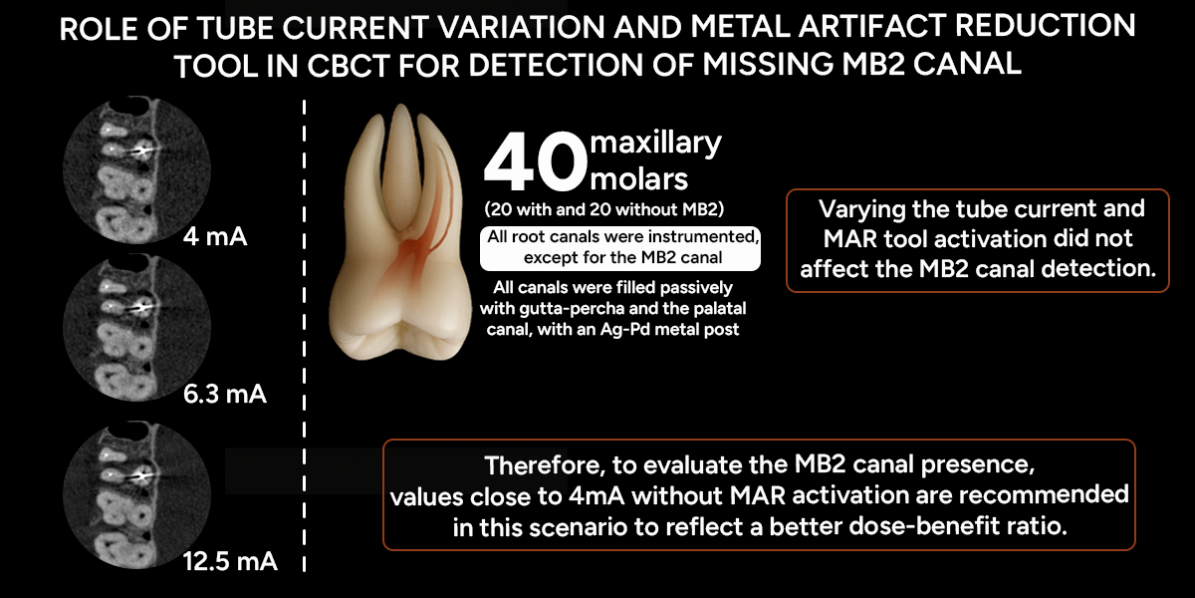

## Introduction

Many treatment failures in maxillary molars can be attributed to the non-localization of the second mesiobuccal (MB2) canals ^1^. Studies indicate that the mesiobuccal root of maxillary first molars frequently contains two canals, with the prevalence of MB2 canals ranging from 30.9% to 96.7%, depending on the studied population and the methodology employed^1^ ^,^ ^2^ ^,^ ^3^. This highlights the remarkable complexity of the root canal system in the mesiobuccal root.

In a context where clinical signs and/or radiographic findings, such as localized pain, increased sensitivity, sinus tract formation, intra- or extraoral edema, and periapical lesions, are observed following endodontic treatment in maxillary molars, it is essential to employ appropriate tools to support the dental surgeon in the investigation of these cases^3^. Therefore, cone-beam computed tomography (CBCT) has contributed substantially to the increased detection of the MB2 canal compared to periapical radiographs^4^. However, the presence of materials with high atomic numbers and high physical density, or the use of lower tube current values, can lead to artifacts and noise, which are limitations of CBCT ^5^ ^,^ ^6^. The presence of these artifacts and noise can modify the density and contrast patterns of the image, altering its quality. Additionally, the former can overlap with regions of interest, impairing diagnosis ^7^.

Due to the beam hardening phenomenon caused by materials such as gutta-percha and metal intracanal posts, streaks and hypodense bands appear on the image, compromising various diagnostic tasks ^8^ ^,^ ^9^. To reduce the effects of artifacts on image quality and, consequently, improve the contrast-to-noise ratio (CNR), a metal artifact reduction (MAR) tool has been developed using post-processing algorithms^7^. Its mode of operation is not publicly available. Still, it seems to act by reducing the variation in gray values by applying a threshold corresponding to the average values^10^.

One of the most practical ways of reducing the dose to patients, thus respecting the ALADAIP (*As Low As Diagnostically Acceptable, being Indication-oriented and Patient-specific*) ^11^ principle, is to reduce the tube current, also known as milliamperage (mA)^12^. However, by reducing this energetic parameter, there is a decrease in the signal-to-noise ratio (SNR), with a risk of loss of image quality due to increased noise in the image, which can compromise various diagnostic tasks ^6^ ^,^ ^12^.

This is particularly important given the necessity for low-dose protocols and the high rate of non-treatment when the MB2 canal is present^1^. Several studies on mA variations in image quality have already been carried out^6^ ^,^ ^13^. However, to date, the individual contributions of tube current and MAR tool activation to the detection of the MB2 canal have not been addressed explicitly in the literature.

Increasing the tube current, which is directly proportional to the radiation dose, typically reduces image noise and improves image quality. In addition, activating the MAR tool aims to reduce metal artifacts by changing gray values, which can potentially increase the CNR. Therefore, this study aimed to assess the impact of tube current variation and the MAR tool on the detection of the MB2 canal in CBCT scans of endodontically treated maxillary molars.

## Materials and methods

The local Research Ethical Committee approved this study under protocol 67662723.0.0000.5418.

### Sample characteristics

**Figure 1:**
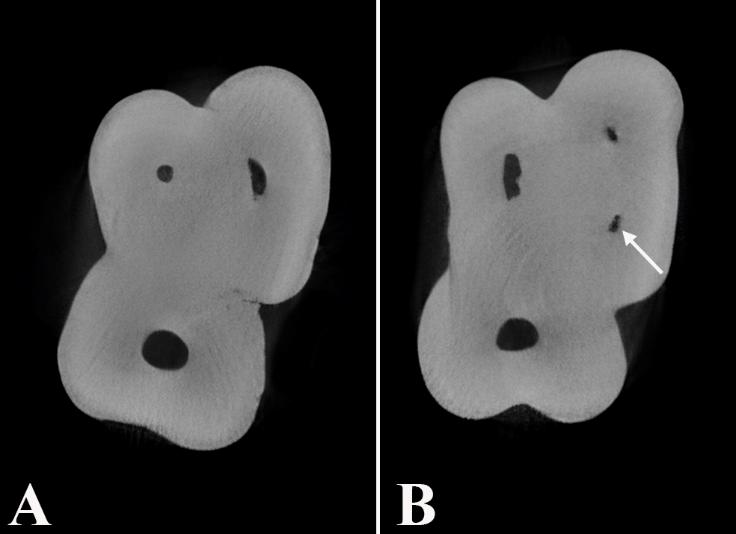
Micro-CT axial images; A. Tooth without the MB2 canal. B. Tooth with the MB2 canal present.

Of the 105 maxillary molars initially collected, which had been extracted for reasons unrelated to this study, 52 were selected based on the analysis of previously obtained periapical radiographs. These radiographs were used to exclude teeth with prior endodontic treatment, supernumerary roots, root resorption, root fractures, or calcified canals. The presence or absence of the MB2 canal was confirmed through microcomputed tomography (micro-CT) analysis, using high-resolution images evaluated in multiple planes. The axial view, in particular, was used to confirm canal identification due to its reliability in visualizing additional canals. This analysis also confirmed the absence of atretic canals and, when present, the independence of the MB2 canal from the MB1 (Figure 1).

For micro-CT acquisition, the Skyscan 1174 device (Bruker, Kontich, Belgium) was set to the following parameters: 50 kV, 800 µA, a 0.5 mm aluminum filter, 0.5° rotation step, one frame averaging, 180° rotation, a scan time of 45 minutes, and a voxel size of 31.03 µm. The device's software was used for image reconstruction and analysis, conducted by two experienced evaluators who reached a consensus on the presence of the MB2 canal.

After micro-CT analysis of the 52 teeth, 20 specimens with an independent MB2 canal were identified. To create a balanced and comparable sample, 20 additional teeth without an MB2 canal were selected, resulting in a final sample of 40 permanent maxillary first molars used for further analysis. After being cleaned with 70% alcohol, each tooth underwent scaling and root planing to eliminate residual soft tissue and calculus, and it was then stored in distilled water.

The root canals of each tooth were instrumented using the WaveOne® reciprocating rotary system (Dentsply Maillefer, Switzerland), with a single-file technique (025.07) applied to the full working length of the canal. Distilled water was used as the irrigating solution throughout the preparation. All root canals were instrumented, except for the MB2 canal in the test group teeth. All canals were filled passively with gutta-percha or, in the case of the palatal canal, with an Ag-Pd (Silver-Palladium) metal post, both in the same dimension as the instruments used for canal preparation. The materials were inserted until the apical portion of the canal was filled, ensuring the material was present throughout the entire canal. The MB2 canal, when present, was not filled with any material to maintain sample integrity and facilitate future analysis.

**Figure 2:**
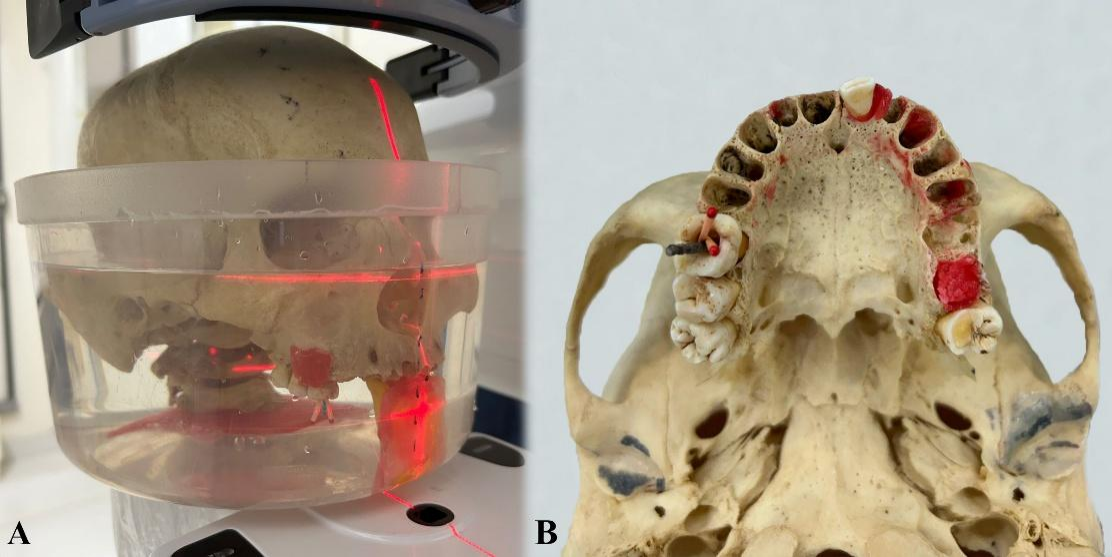
A. The phantom is immersed in water, positioned and centered according to the OP300 reference lines. B. Close-up view of the phantom with the tooth filled with gutta-percha and an intracanal post.

Following tooth preparation, each one was positioned in the corresponding socket-either the right (tooth 16) or left (tooth 26) maxillary first molar socket-of a phantom consisting of a dry human skull and cervical vertebrae C1 and C2 inserted into a plastic container filled with water. The vertebrae were utilized to support the skull and simulate the attenuation of the cervical spine during the CBCT exam; the water was used to simulate the attenuation of X-rays by soft tissues (Figure 2).

### Tomographic acquisitions

The CBCT acquisition was performed using the OP300 Maxio system (Instrumentarium Dental, Tuusula, Finland), and the protocol employed consisted of a field of view (FOV) of 5 x 5 cm, a voxel size of 0.085 mm, and 90 kVp. Three mA settings (4, 6.3, and 12.5 mA) were tested: the manufacturer’s recommended standard (6.3 mA), the lowest (4 mA), and the highest (12.5 mA) available for the smallest voxel size. For each acquisition, images were also reconstructed with the MAR tool activated. The CBCT unit provided dose area products (DAP) of 265, 418, and 830 mGycm², respectively, indicating that the radiation dose increased with higher mA settings.

**Figure 3:**
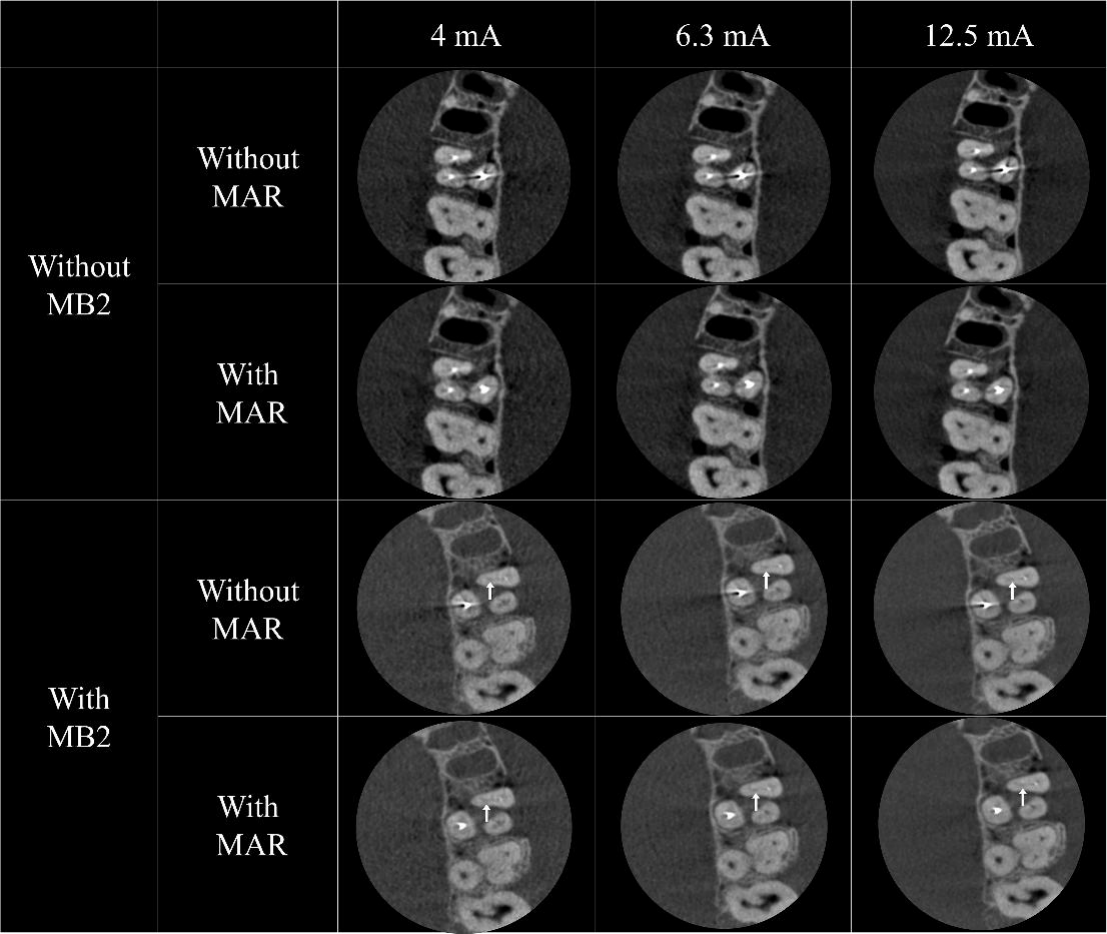
Axial images showing different mA values with or without MAR activation. The white arrow indicates the presence of the MB2 canal.

**Figure 4:**
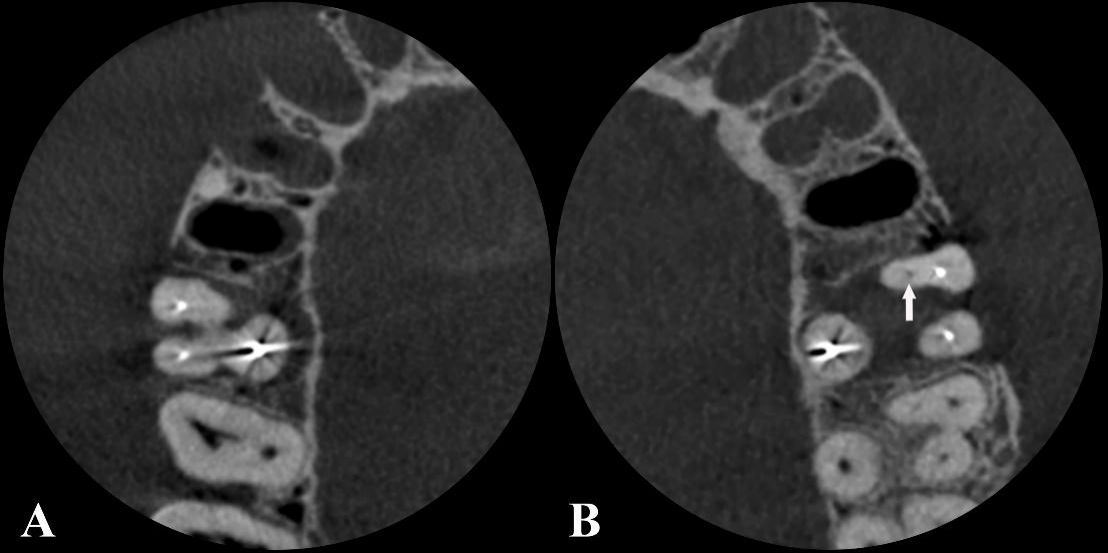
Axial zoom images acquired at 12 mA without MAR tool activation: A. Tooth 16 positioned in the corresponding maxillary socket, showing absence of the MB2 canal. B. Tooth 26 positioned in its corresponding maxillary socket, with the MB2 canal visible (white arrows).

In total, 240 CBCT volumes (40 teeth × 3 mA values × 2 MAR conditions) comprised the images in this study, with each tooth always positioned so that it was centered within the FOV. Figures 3 and 4 display the axial reconstructions of the volumes obtained under different protocols, as well as an approximate view of the CBCT axial reconstruction with and without the MB2 canal, respectively.

### Image evaluation

The five examiners, oral radiologists with at least 2 years of experience in CBCT diagnosis, were previously trained with images that were not part of the sample. The principal investigator instructed them to observe 10 CBCT volumes containing a tooth with an MB2 canal and 10 volumes without an MB2 canal. Subsequently, the enumerated and randomized volumes were submitted for assessment by examiners blinded to the study conditions. The OnDemand3D software (Cybermed, Seoul, Korea) was used on their computers, with a minimum screen size of 15", under reduced lighting conditions. Tools such as brightness, contrast, filters, and zoom adjustment were allowed. Each examiner used a 5-point scale: 1 - absence of the MB2 canal, 2 - probable absence, 3 - an unclear diagnosis, 4 - probable presence of MB2 canal, and 5 - presence of MB2 canal. For analyzing intra- and interexaminer agreement, 25% of the sample was reassessed 30 days after the evaluation.

### Statistical analysis

The area under the receiver operating characteristic curve (AUC), sensitivity, and specificity were calculated for each of the five examiners by confronting their responses with the gold standard. These values were then compared using a two-way Analysis of Variance (ANOVA) to investigate whether the tested factors and their interactions influenced the identification of the MB2 canal. Before performing the ANOVA, the model's underlying assumptions were evaluated. The Shapiro-Wilk test indicated that the residuals followed a normal distribution (p = 0.632, 0.083, and 0.088 for AUC, sensitivity, and specificity data, respectively). Levene's test confirmed the homogeneity of variances across groups (p = 0.349, 0.452, and 0.505 for AUC, sensitivity and specificity data, respectively). These results indicate that the assumptions of the ANOVA were adequately met, ensuring the reliability of the statistical analysis.

Intra- and interexaminer reproducibility was assessed using the weighted Kappa index. Values less than 0.00 indicate poor agreement; 0.00-0.20 indicate slight; 0.21-0.40, fair; 0.41-0.60, moderate; 0.61-0.80, substantial, and 0.81-1.00, almost perfect agreement, as per the classification established by Landis and Koch ^14^. All analyses were performed using SPSS software, version 23.0 (IBM Corp., Armonk, NY, USA), with a significance level set at 5%. The power of the test was 75%, based on the number of repetitions per group, the difference between groups, and standard deviation values.

## Results

Table 1 presents the intra- and interexaminer agreement levels. The intraexaminer agreement was classified as substantial, indicating consistency in the examiners' assessments after re-evaluation. The interexaminer agreement ranged from moderate to substantial, reflecting both the reliability of the results and the challenges faced by the examiners in the diagnostic task.

**Figure 5:**
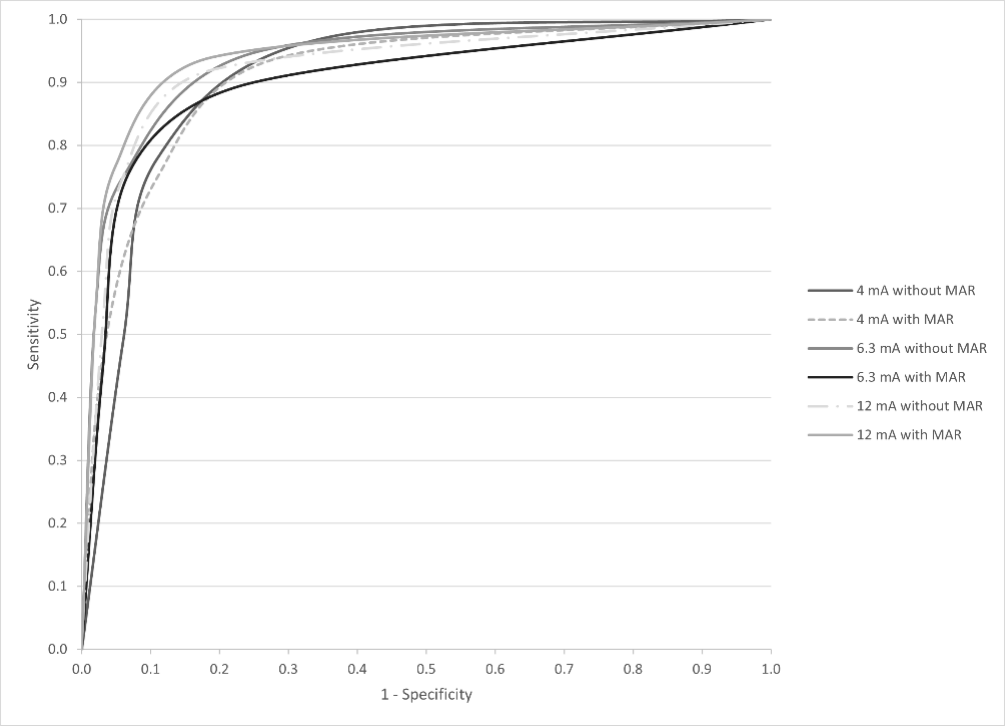
Receiver-operating characteristic curve for the MB2 canal detection under different protocols

Table 2 presents the diagnostic performance metrics, including the AUC, sensitivity, and specificity for MB2 canal identification across different tube current settings, with and without the MAR tool. The AUC values indicated high diagnostic accuracy under all protocols, with no statistically significant differences between tube current settings (p = 0.605) or MAR tool activation (p = 0.728), as shown in Figure 5. Sensitivity showed greater variability compared to AUC and specificity, as reflected by its higher standard deviation values (p mA = 0.985; p MAR = 1.000).

No statistically significant differences were observed in the diagnostic performance across all test conditions, indicating that variations in tube current and MAR tool activation did not impact MB2 canal detection.

**Table 1:** Mean intra- and interexaminer agreement values

Evaluator	1	2	3	4	5
1	0.72	0.62	0.58	0.68	0.63
2		0.78	0.67	0.52	0.49
3			0.63	0.52	0.47
4				0.78	0.60
5					0.75

**Table 2:** Mean (SD) diagnostic values at the different mA settings and activation of the MAR tool

mA	AUC		Sensitivity		Specificity	
	Without MAR	With MAR	Without MAR	With MAR	Without MAR	With MAR
4	0.91 (0.05)	0.91 (0.05)	0.78 (0.12)	0.75 (0.14)	0.89 (0.11)	0.89 (0.11)
6.3	0.93 (0.02)	0.90 (0.04)	0.75 (0.18)	0.76 (0.11)	0.94 (0.07)	0.93 (0.11)
12.5	0.91 (0.03)	0.94 (0.03)	0.75 (0.11)	0.77 (0.10)	0.94 (0.07)	0.95 (0.09)
*p mA; p MAR*	*0.605 ; 0.728*		*0.985; 1.000*		*0.399; 1.000*	

P values according to ANOVA test; AUC (area under the ROC curve); SD (Standard Deviation); mA (milliamperage values); MAR (Metal Artifact Reduction tool)

According to Landis and Koch’s interpretation, intra-observer agreement ranged from 0.63 to 0.78 (substantial), and inter-observer agreement ranged from 0.47 (moderate) to 0.68 (substantial).

However, no statistically significant differences were observed in the diagnostic performance across all test conditions, indicating that variations in tube current and MAR tool activation did not impact MB2 canal detection.

## Discussion

In cases involving previously endodontically treated teeth or teeth with complex root anatomy, in which clinical and two-dimensional radiographic information is insufficient, the American and European guidelines ^15^ ^,^ ^16^ ^,^ ^17^ recommend a small FOV with high-resolution CBCT scans to assist the dental surgeon in the treatment planning. Due to the higher dose inherent in high-resolution scans, our study aimed to evaluate different tube current values to potentially reduce the dose to patients in this context without compromising the diagnosis.

The use of low-dose protocols in CBCT is an approach that aims to minimize the patient's exposure to radiation without compromising the image quality required for diagnosis and treatment planning ^18^ ^,^ ^19^. These inconsistent X-ray beam attenuation values can degrade the image by increasing noise^5^ ^,^ ^20^. Even with a theoretical improvement in the CNR^21^, increasing the tube current did not improve the detection of the MB2 canal in our study. Therefore, reducing the tube current is recommended in this context.

With smaller FOV sizes and lower tube current values, the patient dose is reduced, confirmed by the dose area product (DAP) provided by the OP300 Maxio manual. The DAP for 4, 6.3, and 12.5 mA was 265, 418, and 830 mGycm², respectively, maintaining a directly proportional relationship between this energetic factor and the dose received by the patient.

McGuigan et al. ^21^ studied to determine a threshold dose that complied with the ALARA principle for detecting the MB2 canal using CBCT. Comparing two different CBCT scanners under parameters of 70, 80, and 90 kVp and 3 to 9 mA, the authors concluded that the threshold dose would be just over 200 mGycm², which is lower than the DAP in our study (265 mGycm²) when 4 mA was used. They also concluded that using high-resolution protocols did not improve the visualization of complex root canals.

However, a crucial methodological difference is that McGuigan et al. only evaluated teeth with the MB2 canal. Therefore, the examiners evaluated the canal complexity rather than detection. Additionally, none of the teeth had high-density intracanal material. In our study, the canals were filled with gutta-percha or an Ag-Pd post (except for MB2), similar to a clinical scenario. The presence of these materials produces additional artefacts and contrast variations, possibly making MB2 canal detection more difficult. This difference in diagnostic difficulty, rather than scanner performance per se, likely accounts for the divergent results between the two studies.

Various studies have been carried out to assess the influence of CBCT tube current in different diagnostic tasks^6^ ^,^ ^9^ ^,^ ^13^ ^,^ ^22^ ^,^ ^23^. In cases of vertical root fractures (VRF) with adjacent implants and the use of MAR^9^, the use of 4mA without the MAR tool is recommended, as there was no difference in fracture assessment using higher parameters, as evidenced by our results.

Another study using different intracanal materials for VRF detection ^23^ concluded that lower mA values (4 mA) could be used when no material is present or when a glass fiber post is used. However, higher mA values (8 mA) were recommended when gutta-percha or metal posts were present. This contrasts with the current study, which used gutta-percha and an Ag-Pd post on the same multi-rooted tooth. This can be explained by the intracanal material in single-rooted teeth, as observed in previous research, which may have had a more significant influence on the artifact. Additionally, the structures subject to each investigation varied in size; for example, a root fracture is more radiographically thin than a root canal ^24^.

Lower tube current values lead to a decreased SNR in the image ^25^, and the MAR tool, by enhancing the CNR, can improve image quality when metal structures are present ^7^ ^,^ ^10^ ^,^ ^25^. Nevertheless, using MAR at any of the mA values did not result in a significant difference in our study. This finding is consistent with previous investigations on the influence of MAR ^9^ ^,^ ^13^ ^,^ ^26^, which also reported no significant variations in effectiveness across different diagnostic tasks.

In a previous study, the type of intracanal material did not influence MB2 canal detection using 6.3 mA for the same CBCT device. However, using gutta-percha and Ag-Pd posts is justified to simulate the clinical context better, as materials with high physical density and high atomic number may be present in a patient's oral cavity during oral rehabilitation. These materials can obscure the region of interest and mimic conditions of endodontic failure if the patient's symptoms persist^7^ ^,^ ^27^.

The moderate intra- and inter-examiner agreement values, compared to the AUC, sensitivity, and specificity values, could be attributed to several factors. Besides the inherent difficulty of this diagnosis, the use of a 5-point scale may have introduced additional variability and subjectivity. For instance, even if different examiners correctly identified the presence of the canal, their ratings might have varied between 4 and 5, thus reducing inter-examiner agreement. Our findings are consistent with previous literature^26^ ^,^ ^28^, which reported similar variability in intra- and inter-examiner agreement for MB2 canal detection.

It is essential to acknowledge the inherent limitations of an ex vivo study, including the absence of clinical data, the lack of radiographic signs in the alveolar bone, and the potential for artifacts due to patient movement. However, the possibility of standardized positioning and repeated exposures, along with the total control of variables, increased the reliability of our results. Future clinical studies are recommended to test the use of lower tube current values and other tomographic systems on specimens with varying root anatomy.

Therefore, the findings of this ex vivo study demonstrated that varying the tube current and using the MAR tool did not impact the detection of the MB2 canal. In alignment with the radioprotection principles outlined by ALADAIP, it is advised that a lower tube current value (4 mA) be considered when identifying this canal, which may enhance the dose-benefit ratio. Additionally, considering the absence of any diagnostic advantage and the increased time required for reconstruction of the examination, the use of the MAR tool is not recommended for this specific diagnostic task.
